# Prognostic Value of SUVmax Measured by Pretreatment Fluorine-18 Fluorodeoxyglucose Positron Emission Tomography/Computed Tomography in Patients with Ewing Sarcoma

**DOI:** 10.1371/journal.pone.0153281

**Published:** 2016-04-21

**Authors:** Jae Pil Hwang, Ilhan Lim, Chang-Bae Kong, Dae Geun Jeon, Byung Hyun Byun, Byung Il Kim, Chang Woon Choi, Sang Moo Lim

**Affiliations:** 1 Department of Nuclear Medicine, Soonchunhyang University Bucheon Hospital, Bucheon, Korea; 2 Department of Nuclear Medicine, Korea Institute of Radiological and Medical Sciences, Seoul, Korea; 3 Molecular Imaging Research Center, Korea Institute of Radiological and Medical Sciences, Seoul, Korea; 4 Department of Orthopedic Surgery, Korea Institute of Radiological and Medical Sciences, Seoul, Korea; Faculté de médecine de Nantes, FRANCE

## Abstract

**Aim:**

The aim of this retrospective study was to determine whether glucose metabolism assessed by using Fluorine-18 (F-18) fluorodeoxyglucose (FDG) positron emission tomography/computed tomography (PET/CT) provides prognostic information independent of established prognostic factors in patients with Ewing sarcoma.

**Methods:**

We retrospectively reviewed the medical records of 34 patients (men, 19; women, 15; mean age, 14.5 ± 9.7 years) with pathologically proven Ewing sarcoma. They had undergone F-18 FDG PET/CT as part of a pretreatment workup between September 2006 and April 2012. In this analysis, patients were classified by age, sex, initial location, size, and maximum standardized uptake value (SUVmax). The relationship between FDG uptake and survival was analyzed using the Kaplan-Meier method with the log-rank test and Cox’s proportional hazards regression model.

**Results:**

The median survival time for all 34 subjects was 999 days and the median SUV by using PET/CT was 5.8 (range, 2–18.1). Patients with a SUVmax ≤ 5.8 survived significantly longer than those with a SUVmax > 5.8 (median survival time, 1265 vs. 656 days; p = 0.002). Survival was also found to be significantly related to age (p = 0.024), size (p = 0.03), and initial tumor location (p = 0.036). Multivariate analysis revealed that a higher SUVmax (p = 0.003; confidence interval [CI], 3.63–508.26; hazard ratio [HR], 42.98), older age (p = 0.023; CI, 1.34–54.80; HR, 8.59), and higher stage (p = 0.03; CI, 1.21–43.95; HR, 7.3) were associated with worse overall survival.

**Conclusions:**

SUVmax measured by pretreatment F-18-FDG PET/CT can predict overall survival in patients with Ewing sarcoma.

## Introduction

Ewing sarcoma is the second most common primary bone tumor [1]. Owing to the use of multimodal therapy in the form of chemotherapy and surgery, with or without radiotherapy, survival has improved to 60–70% [2]. Nevertheless, 30–40% of patients will develop local and/or distant recurrent disease typically between 2 and 10 years after diagnosis [3].

Survival of patients with this malignancy is related to the presence of metastatic disease, tumor size and histologic response after chemotherapy [4,5]. Because Ewing sarcoma is an aggressive bone or soft tissue malignancy which usually metastasizes to the lungs or bone marrow [6,7], an extensive workup is important to define the tumor stage accurately including lymph node involvement and the presence of distant metastases. Subgroups of patients that may benefit from aggressive therapy (surgery, chemotherapy, and radiotherapy) should be accurately identified. Accordingly, prognostic factors that permit the identification of patients likely to benefit from treatment are clinically relevant.

Traditional imaging methods for evaluation of bone and soft tissue sarcomas include plain radiography, magnetic resonance imaging (MRI) of the primary tumor, computed tomography (CT) and bone scintigraphy to detect regional or distant metastases [8,9].

Fluorine-18 (F-18) fluorodeoxyglucose (FDG) positron emission tomography (PET)/CT is a relatively recent, noninvasive imaging technique based on the increased glucose uptake of malignant cells [10,11]. F-18 FDG PET can detect tumors earlier than conventional imaging, and can evaluate the aggressiveness of the tumor and predict prognosis based on the increased glucose uptake by malignant cells. As Ewing sarcoma is a malignant tumor with a tendency to early systemic spread, this imaging tool could prove to be useful in identifying and selecting patients with disseminated disease not amenable to curative resection.

Recent studies using F-18 FDG PET/CT for Ewing sarcoma were focused on the evaluation of staging [12], response monitoring [13], and detection of distant metastasis [14] or recurrence [15]. However, few studies have assessed FDG uptake of pretreatment F-18 FDG PET/CT as a prognostic indicator in patients with Ewing sarcoma.

Therefore, the aim of this study was to investigate the prognostic value of F-18 FDG PET/CT in patients with Ewing sarcoma.

## Materials and Methods

### Patient population

The institutional review board of Korea Cancer Center Hospital approved this retrospective study. The requirement to obtain informed consent was waived and patient’s records were anonymized prior to analysis. Thirty-four consecutive patients with Ewing sarcoma who underwent pretreatment F-18 FDG PET/CT and received surgery following adjuvant chemotherapy as a treatment protocol between September 2006 and April 2012 were enrolled in our retrospective study. The presence of Ewing sarcoma was histologically proven in all 34 patients using specimens obtained at surgery. The pathologic diagnosis and classification of the tumors were made according to the American Joint Committee on Cancer (AJCC, 7^th^ edition) staging system. The clinical and pathologic records of each patient were reviewed, and the following information was gathered: age, sex, maximum standardized uptake value (SUVmax), initial location, tumor size, and AJCC stage.

### FDG PET/CT imaging

All patients fasted for at least 6 h before the administration of ^18^F-FDG, and 10–12 mCi (370–444 MBq) of ^18^F-FDG was injected intravenously 1 h prior to imaging. The blood sugar levels of all patients were measured prior to the injection of ^18^F-FDG. A non-enhanced low-dose CT scan was obtained for attenuation correction because all patients had already undergone contrast-enhanced abdominopelvic CT before the FDG PET/CT scan. The CT portion of the Discovery LS consists of a multidetector helical scanner (LightSpeed Plus; General Electric Medical Systems), and the Biograph 6 consists of a 6-slice CT. Imaging parameters were as follows for acquisitions in 5–7 bed positions: 140 kV, 80 mA, 0.8 s per CT rotation, a pitch of 6, a table speed of 22.5 mm/s, 722.5–1011.5 mm coverage, 130 kV, 30 mA, 0.6 s per CT rotation, a pitch of 1.5, a 31.9–37-s acquisition time for the Discovery LS, and a 20.89-s acquisition time for the Biograph 6. The CT scans were performed before the PET scans. The CT tube current was adjusted according to patient weight. The CT data were resized from a 512 × 512 matrix to a 128 × 128 matrix to match the PET data, and a CT transmission map was generated to fuse the images. PET emission data were acquired in 5–7 bed positions, typically from the base of the skull through the upper thigh. Emission data were acquired for 6 min in each bed position. Each bed had 35 (Discovery LS) or 39 (Biograph 6) scanning planes with a 14.6-cm (Discovery LS) or 16.2-cm (Biograph 6) longitudinal field-of-view and a 1-slice overlap. PET images were reconstructed using CT for attenuation correction with the ordered-subsets expectation maximization algorithm (2 iterations and 8 subsets) and a 5-mm Gaussian filter using a 128 × 128 matrix.

For inter-scanner comparison of the SUV values, the measurement of radioactivity is the most important, because the SUV value has a linear relationship with the radioactivity. Therefore, we performed an experiment to evaluate the variability in the radioactivity measurements from both of the PET/CT cameras, using a pie-shaped phantom. In this experiment, we used the same parameters for acquisition and reconstruction of images as used in the human study. We obtained pie-shaped images with 6 sectors reflecting different F-18 radioactivity levels (0, 7000, 14,000, 28,000, 57,000, and 114,000 MBq/ml), and placed 6 small circular regions of interest on each sector to get pixel values in MBq/ml. The values of radioactivity from the images were compared and showed that the variable difference was <5% in quantitative measurements between the 2 PET/CT cameras (estimated regression slope = 0.98; *r*^2^ = 0.99; p = 0.001).

### Image analysis

All PET/CT scans were examined retrospectively by 2 experienced nuclear medicine physicians on an interactive computer display using fusion software (Xeleris; General Electric Medical Systems; and Syngo; Siemens Medical Solutions). This software allows review of PET, CT, and fused data using transaxial, sagittal, and coronal displays. To perform a quantitative analysis, the SUV was calculated using the following equation: decay-corrected activity [kBq]/ml of tissue volume/injected F-18 FDG activity [kBq]/g of body mass. For the SUV analysis, a circular region of interest was placed over the area of maximal focal FDG uptake suspected to be a tumoral focus, and the maximal values were obtained.

### Statistical analysis

The statistical analysis was performed using Medcalc software v. 11.3. All values are expressed as the mean ± the standard deviation (SD). The following statistical analyses of potential prognostic factors were performed for all 86 patients with PGL. Patients were stratified and analyzed in a univariate analysis, using age, sex, site of primary lesion, tumor size, AJCC stage, and the SUVmax of the primary lesion. Patients were classified into low SUVmax and high SUVmax subgroups by an ROC curve analysis. Survival time was defined as the time from pre-treatment FDG PET /CT scan to the date of the detection of death or to the date of the last follow-up visit at our medical center. Overall cumulative survival was analyzed by the Kaplan–Meier method, and differences in survival between subgroups were compared using a log-rank test.

Null hypotheses of no difference were rejected if *p*-values were less than 0.05. Variables with *p* < 0.05 in the univariate analysis of factors affecting survival were included in a subsequent multivariate analysis using Cox’s proportional hazard model. To evaluate multi-collinearity between histologic types, the chi-squared test was applied before the multivariate analyses. Moreover, the Mann–Whitney *U* test, Fisher’s exact test, and the chi-squared test were employed to compare clinicopathologic factors and FDG PET/CT parameters between patients in the higher and lower SUVmax groups.

## Results

### Patient characteristics

The patients’ characteristics are detailed in Table 1. The mean age of the patients was 14.5 ± 9.7 years (range, 3–43 years; 19 men and 15 women). The overall median survival time was 999 days and the median SUVmax was 5.8 (range, 2–18.1). The mean duration of clinical follow-up was 36.7 ± 3.7 mo (median, 33 mo; range, 6–82 mo). The median tumor size for all of the patients was 8 cm (range, 2.6–24.0 cm). Stages IIA, IIB, and IV disease was found in 17, 5, and 12 patients, respectively. Of the initial tumor locations, 14 were in the extremity bones (humerus, 2; tibia, 4; femur, 8), 8 were in the extra-extremity bones (spine, 1; pelvis, 4; scapula, 2; mandible, 1), and 12 were in extraskeletal lesions (thigh muscle, 5; arm muscle, 2; lungs, 2; bowel, 1; chest wall and ovary, 2).

**Table 1:** Patient’s Characteristics for Groups With SUVmax.

Variable factors	Low SUV (≤5.8)	High SUV (>5.8)	p value
SUVmax* (mean ± SD)	3.6 ± 1.0	9.4 ± 3.5	<0.001
Median survival (days)	1236	627	0.004
Age (mean ± SD, years)	14.9 ± 7.7	19.3 ± 11.4	0.277
Sex			
Male	11	8	0.489
Female	6	9	
Stage			
IIA	12	5	
IIB	2	3	0.047
IV	3	9	
Initial location			
Extremity bones	9	5	
Extra-extremity bones	1	7	0.050
Extraskeletal lesions	7	5	
Size (cm)			
≤8	3	7	0.179
>8	14	10	

*SUVmax = maximum standardized uptake value

### Comparisons of survival by SUVmax

The cutoff value for SUVmax in all 34 patients was 5.8 (17 and 17 patients had a SUVmax <5.8 and ≥5.8, respectively), which was the median value in agreement with the results of receiver operating characteristic curve analysis. The median survival time for patients with a SUVmax ≤ 5.8 was 1236 days compared to 627 days for those with a SUVmax > 5.8 (p < 0.004). These 2 groups significantly differed with regard to stage (p = 0.047) and initial tumor location (p = 0.05). Patient groups with stage IV had a higher SUVmax than those with stage II. We found a tendency for larger tumor size in the patient group with a higher SUVmax.

### Survival analysis

A Kaplan-Meier curve was constructed for the 2 SUVmax groups. In univariate analysis, SUVmax (p = 0.0002), tumor stage (p = 0.01), age (p = 0.007), initial tumor location (p = 0.03), and tumor size (p = 0.03) showed a significant relationship with overall survival (Table 2).

**Table 2:** Univariate analysis for overall survival.

Variable	No.	Median survival (95% CI)	p value
Age (years)			
≤20	24	1071 (730.67–1692.11)	0.007
>20	10	651 (293.19–953.52)	
Sex			
male	21	1048 (655.12–1248.86)	0.220
female	13	756 (618.52–1746.93)	
SUVmax*			
≤5.8	17	1236 (973.85–1756.38)	< 0.001
>5.8	17	627 (443.09–1046.13)	
Stage			
IIA	17	1236 (656.51–1980.34)	
IIB	5	1097 (402.34–1750.85)	0.010
IV	12	706 (407.21–1035.04)	
Initial tumor location			
Extremity bones	14	1152 (644.53–1987.85)	
Extra-extremity bones	8	794 (302.31–1574.81)	0.030
Extraskeletal lesions	12	880(621.20–1611.53)	
Size (cm)			
≤8	24	1037 (730.67–1393.27)	0.030
>8	10	623 (405.29–1611.75)	

* SUVmax = maximal standardized uptake value

**Fig 1:**
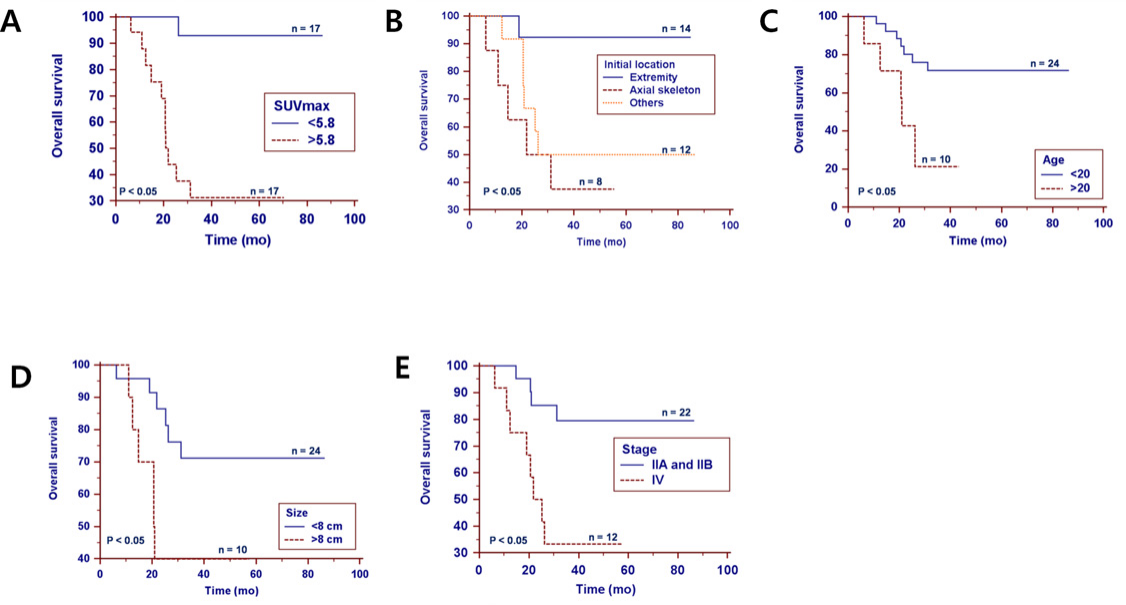
Kaplan–Meier survival curves depicting overall survival. (A) SUVmax, (B) initial tumor location (b; other locations of the primary tumor were the thigh muscles, arm muscles, lungs, bowel and soft tissues in 5, 2, 2, 1, and 2 patients, respectively), (C) age, and (D) tumor size and (E) stage.

In multivariate analysis, SUVmax (p = 0.003), stage (p = 0.03), and age (p = 0.023) were found to be independent predictors of overall survival. In particular, the hazard ratios for SUVmax, age, and stage were 42.9 (95% CI, 3.63–508.26), 8.6 (95% CI, 1.34–54.80), and 7.3 (95% CI, 1.21–43.95), respectively (Table 3 and Fig 1).

**Table 3:** Multivariate Cox proportional hazards models.

Covariable factors	Hazard ratio	95% Confidence intervals	p value
SUVmax*	42.9	3.63–508.26	0.003
Stage	7.3	1.21–43.95	0.030
Age (years)	8.6	1.34–54.80	0.023
Initial location	1.7	0.61–4.52	0.322
Tumor size (cm)	4.65	0.67–32.24	0.121

* SUVmax = maximal standardized uptake value

## Discussion

Our study evaluated the association between FDG uptake of the primary tumor and prognosis in patients with Ewing sarcoma, and demonstrated that the SUVmax of the primary tumor was an independent prognostic factor.

Univariate analysis revealed that survival was significantly influenced by SUVmax, age, stage, tumor size, and initial tumor location. In multivariate analysis, SUVmax, stage, and age were significantly associated with overall survival. The HR for the higher SUVmax scores was 42.9 times that of the lower SUVmax groups and was independent of other prognostic factors. The HR of SUVmax was higher than that of age and stage, which are well known prognostic factors for Ewing sarcoma.

According to our results, SUVmax calculated from F-18 FDG PET is a strong independent prognostic parameter of Ewing sarcoma, allowing accurate identification of patients who will benefit from intensive anticancer treatment at different stages of the disease.

In a previous study, Hawkins et al. reported that a SUV2 < 2.5 (measured after neoadjuvant chemotherapy) is predictive of progression-free survival, independent of initial disease stage, but SUV1 (measured before treatment) was not found to be a statistically significant prognostic factor [16]. They focused on treatment response or status using post-treatment parameters (SUV2 and histologic response) and analyzed progression-free survival. However, small variations in patient numbers had the potential to reduce the statistical power of the multivariate analysis. In contrast, in our study, we focused on the pretreatment status and found that pretreatment SUVmax is an independent predictor of overall survival (baseline metabolic parameter without any treatment).

In another study, Eary et al. reported that multivariate analyses showed that SUVmax is a statistically significant independent predictor of sarcoma patient survival, and tumors with a higher SUVmax had a significantly poorer prognosis [17]. This study included histologically heterogeneous sarcomas, but our study included only pathologically proven Ewing sarcomas.

Andersen et al. also reported that pretherapeutic SUVmax using FDG PET/CT demonstrates independent properties beyond histologic grading for prediction of survival and this result supports our research [18].

In musculoskeletal tumors, FDG PET/CT plays an important role in initial staging, detecting distant metastasis or early recurrence, restaging, monitoring of treatment response and it is useful in subsequent treatment strategy [19–21].

Van Maldegem et al. reported that a tumor size > 8 cm and the presence of metastasis appeared to be strong predictors of a negative outcome [22]. Although this study did not use FDG PET, their results corresponded with ours.

Tumor size had a significant association with survival in univariate analysis, but did not have an independent effect in multivariate analysis (p = 0.120). This may have been due to the small number of enrolled patients in our study.

F-18 FDG PET/CT has the advantage of being able to detect early incidental or unexpected distant metastasis during initial staging, which could influence clinical decisions, and predict the resectability and prognosis of patients with Ewing sarcoma.

A major limitation of our retrospective study is the relatively small number of patients included. In addition, because it was a single-center study with a retrospective design, selection bias was inherent and the general applicability of our study may therefore be limited. Finally, differences in SUVmax among different PET scanners might comprise a limitation to the application of these results in other institutions. A future well-organized large-scale prospective study is warranted to overcome this limitation.

In addition, we can not evaluate a histologic response which is known prognostic factor of Ewing sarcoma, because our enrolled patients did not receive a neoadjuvant chemotherapy.

In conclusion, SUVmax measured by using F-18 FDG PET/CT was found to be significantly related to overall survival and could be useful to predict the prognosis of patients with Ewing sarcoma.

## Conclusions

The SUVmax measured by pretreatment ^18^F-FDG PET/CT can predict the overall survival of patients with Ewing sarcoma. We anticipate that this prognostic stratification based on SUVmax will be important for predicting overall survival.

## Supporting Information

S1 File Raw datas of Ewing sarcoma patients. (XLSX)

S2 File Statistics using Medcalc software. (MC1)
